# Decreased estimated glomerular filtration rate predicts long-term recurrence after catheter ablation of atrial fibrillation in mild to moderate renal insufficiency

**DOI:** 10.1186/s12872-021-02320-y

**Published:** 2021-10-21

**Authors:** Jing Zheng, Deling Zu, Keyun Cheng, Yunlong Xia, Yingxue Dong, Zhenyan Gao

**Affiliations:** 1 Department of Cardiology, The Quzhou Affiliated Hospital of Wenzhou Medical University, Quzhou People’s Hospital, Quzhou, 324000 Zhejiang China; 2 Department of Cardiology, The First Affiliated Hospital of Dalian Medical University, Dalian, 116000 Liaoning China

**Keywords:** Estimated glomerular filtration rate, Atrial fibrillation, Catheter ablation, Recurrence, Mild to moderate renal insufficiency

## Abstract

**Background:**

Catheter ablation is an established therapy for atrial fibrillation (AF), but recurrence after ablation remains a great challenge. Additionally, little is known about the effect of renal function on the efficiency of AF ablation. This study aimed to evaluate the predictors of the prognosis of catheter ablation for AF, especially the effect of renal function.

**Methods:**

A total of 306 drug-refractory symptomatic patients with AF who underwent first-time catheter ablation were enrolled in the present study. Individuals underwent circumferential pulmonary vein isolation for paroxysmal AF and stepwise ablation for persistent AF.

**Results:**

The follow-up time was 27.2 ± 19.5 months, 202 patients (66.01%) were free of atrial tachyarrhythmia (non-recurrence group), and the other 104 patients experienced recurrence (recurrence group). The recurrence group had a larger left atrial diameter (LAD) and left atrial volume (LAV), a higher LAV index (LAVI) (both, *p* < 0.01), and a lower estimated glomerular filtration rate (eGFR) (53.5 ± 14.4 vs. 65.5 ± 13.3 ml/min/1.73^2^, *p* < 0.001) and creatinine clearance rate (CCr) (85.2 ± 26.1 vs. 101.5 ± 29.4 ml/min, *p* < 0.05). Multivariate logistic regression indicated both eGFR (*p* = 0.002) and LAVI (*p* < 0.001) as independent associated factors for long-term recurrence after single catheter ablation; multivariate Cox proportional hazard regression with backward feature selection identified both eGFR (HR: 0.93, 95% CI: 0.91–0.95, *p* < 0.001) and LAVI (HR: 1.32, 95% CI: 1.25–1.40, *p* < 0.001) as independent prognostic factors for recurrence when adjusting other clinical variables.

**Conclusions:**

Decreased eGFR and elevated LAVI may facilitate the long-term recurrence of atrial tachyarrhythmia after catheter ablation for AF.

## Background

Atrial fibrillation (AF) is a common arrhythmia that accounts for a significantly increased risk of stroke and all-cause mortality [1–3]. Catheter ablation has evolved over the past decade and has been demonstrated as an established therapy for paroxysmal AF and persistent AF [4]. However, recurrence after ablation is still a great challenge. In clinical work, standardized and reasonable evaluation of ablation prognosis is beneficial to optimize the selection of individualized treatment and reduce additional medical expenditure. There are several identical risk factors between AF and renal insufficiency, such as hypertension, diabetes mellitus, and age [5]. Additionally, a higher prevalence of AF has been reported among patients with different stages of chronic renal disease [6]. However, the effect of renal function on the efficiency of AF ablation has not been well elucidated. The present study aimed to retrospectively analyse the risk factors for recurrence after first-time catheter ablation of AF, with special attention to the role of renal function.

## Materials and methods

### Study population

This is a single-center retrospective study aimed to assess the predictors of the long-term prognosis of AF catheter ablation. The present study enrolled 306 AF patients who underwent first-time catheter ablation between January 2008 and April 2013 in the First Affiliated Hospital of Dalian Medical University. The inclusion criteria were as follows: age between 18 and 80 years, symptomatic AF despite the use of at least one antiarrhythmic drug, prior attempts of electrical cardioversion, and severe adverse events on rhythm-control drugs; exclusion criteria were defined as severe cardiac valvular diseases, left atrial diameter (LAD) > 50 mm, left atrial thrombus, known bleeding diathesis, prior ablation for AF, and other severe comorbidities resulting in intolerance of perioperative antiarrhythmic/anticoagulation drugs. As our design is a retrospective study, we therefore performed post-hoc power analyses through G*power software with parameters by default (effect size of 0.5 and α of 0.05). The estimated power for our study design was 0.99, indicating our study has a high probability of detecting a real effect. All patients signed an informed written consent form to the study protocol that was approved by Ethics committee of First Affiliated Hospital of Dalian Medical University.

### Pre-procedure management

For paroxysmal AF, low-molecular-weight heparin was administered in the pre-procedure period. For persistent AF, effective anticoagulation therapy with warfarin was performed targeting an international normalized ratio (INR) of 2 to 3 for more than 3 weeks. Warfarin was discontinued 5 days before the procedure and substituted with low-molecular-weight heparin Moreover, antiarrhythmic agents, except amiodarone, were discontinued for at least 5 half-lives before the procedure. Renal function was evaluated by the estimated glomerular filtration rate (eGFR) and creatinine clearance rate (CCr) 1–2 days before the procedure. eGFR was calculated by the abbreviated Modification of Diet in Renal Disease formula [7], and CCr was calculated by the Modification of the Cockcroft–Gault formula [8]. All patients underwent transthoracic echocardiography performed by two experienced senior cardiac sonographers. Left atrial volume (LAV) was assessed offline with Simpson’s method using apical four-chamber and apical two-chamber views at ventricular end-systole [9] and indexed to body surface area calculated by the DuBois formula [10]. CHADS_2_ (congestive heart failure, hypertension, age ≥ 75 years, type 2 diabetes, and previous stroke or transient ischaemic attack [doubled]) [11], CHA_2_DS_2_-VASc (congestive heart failure, hypertension, age ≥ 75 years [doubled], type 2 diabetes, previous stroke, previous stroke or transient ischaemic attack [doubled], vascular disease, age 65 to 75 years, and sex category) [10] and R_2_CHADS_2_ (renal insufficiency [doubled], congestive heart failure, hypertension, age ≥ 75 years, type 2 diabetes, and previous stroke or transient ischaemic attack [doubled]) [12] scores were calculated for each individual.

### Electrophysiological study and catheter ablation

After exclusion of intracardiac thrombi by transesophageal echocardiography and assessment of pulmonary veins by CT scanning, catheter ablation was performed according to the HRS/EHRA/ECAS 2007 Consensus Statement on Catheter and Surgical Ablation of AF [13]. Circumferential pulmonary vein isolation (CPVI) was performed for paroxysmal AF. and stepwise ablation was performed for persistent AF. If AF or atrial tachycardia continued despite the wide ablation above, pharmacological/electrical cardioversion was performed afterwards.

### Post-procedure management

After ablation, oral anticoagulation with warfarin was continued for at least 3 months, and subcutaneous low-molecular-weight heparin injections were discontinued after targeting INR. Amiodarone was administered to persistent AF patients routinely with oral doses of 600 mg/day for 1 week and 200 mg/day for 3 months after ablation.

### Follow-up

After discharge from the hospital, patients were followed up systematically at 1, 3, 6 and 12 months and then every 6 months in the outpatient department, including 24-h Holter recording and 12-lead electrocardiogram. Patients were encouraged to report palpitations and any other symptoms suggestive of tachycardia outside follow-up visits. Recurrence was defined as atrial tachyarrhythmias sustained for more than 30 s beyond a blanking period of 3 months [13], including AF, atrial flutter or atrial tachycardia.

### Statistical analyses

Descriptive statistics are presented as the mean ± standard deviation for continuous variables and as frequency (percentages) for categorical variables. Group comparisons were performed using the t-test or χ^2^-test, as appropriate. Multivariate logistic regression analysis was used to identify the associated factor for recurrence [15]. Receiver operating characteristic (ROC) curves were generated, and area under the curve (AUC) value was calculated to compare and evaluate the predictive performance of the independent predictors of AF recurrence after ablation. Survival analysis was performed using the R package “*survival*”. Specifically, a Kaplan–Meier curve was generated for survival rates of patients with difference detection of log-rank test. Cox proportional hazards regression model was used to calculate hazard ratios (HRs) and 95% confidence intervals (CIs) regarding recurrent-free survival (RFS). Specifically, we performed univariate Cox proportional hazards regression to identified RFS-associated clinical variables; variables significant in univariate analysis (*p* < 0.05) were involved in the multiple Cox proportional hazards regression model, which was further optimized through stepwise regression of backward elimination to detect independent prognostic predictors of recurrence of atrial tachyarrhythmia after catheter ablation for AF. For all statistical analysis, a two-tailed *p-*value less than 0.05 was considered statistically significant. Statistical analysis was conducted using SPSS 24.0, MedCalc 7.3 and R (version 4.0.2).

## Results

### Baseline characteristics and procedure data

**Fig. 1:**
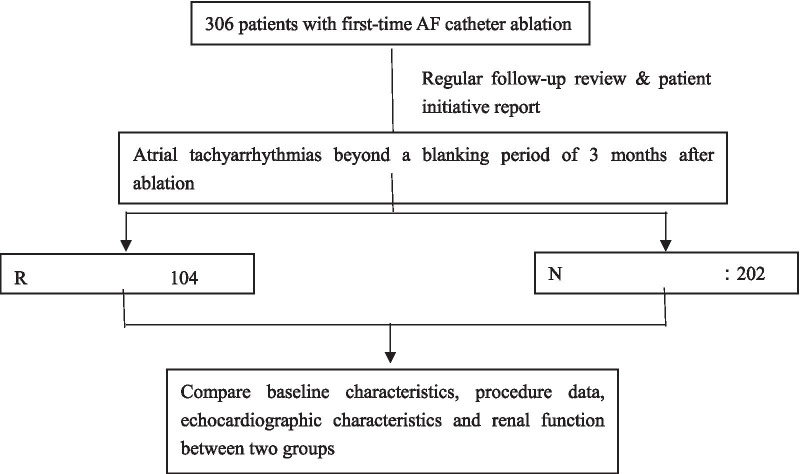
Flowchart of the study. AF indicates atrial fibrillation

A total of 306 patients were enrolled in the present study, including patients with persistent AF (n = 120) underwent stepwise ablation, and patients with paroxysmal AF (n = 186) underwent CPVI (Fig. 1). The mean age was 56.7 ± 10.4 years. In total, 226 cases were male (73.9%). There were 30 patients with CKD G1, 183 patients with CKD G2, 39 patients with CKD G3b, 53 patients with CKD G3a and 1 CKD G4 in our study. Patients included in this study were mainly mild to moderate renal insufficiency (CKD G2-CKD G3a). Following up 27.2 ± 19.5 months after a single procedure, 104 patients (34.0%) experienced recurring atrial tachyarrhythmias (recurrence group), with 40.0% for persistent AF patients and 30.1% for paroxysmal AF patients. In addition, 202 patients maintained sinus rhythm without antiarrhythmic drugs (non-recurrence group). There were no significant differences in age, male sex, hypertension, diabetes mellitus, AF type, coronary heart disease, ischaemic stroke, CHADS_2_, CHA_2_DS_2_-VASc and R_2_CHADS_2_ scores, or procedure data (procedural time, X-ray exposure time, ablation time) between the two groups (Table 1).

**Table 1:** Baseline characteristics between recurrence and non-recurrence groups

	Recurrence Group (n = 104)	Non-recurrence Group (n = 202)	*P* value
Age, years	57.0 ± 10.9	56.6 ± 10.1	0.464
Female, n (%)	31 (29.8)	49 (24.3)	0.295
History of AF, years	3.0 ± 1.5	2.6 ± 2.3	0.848
Persistent AF, n (%)	48 (46.2)	72 (35.6)	0.065
Hypertension, n (%)	46 (44.2)	83 (41.1)	0.598
Diabetes mellitus, n (%)	6 (5.8)	23 (11.4)	0.112
Coronary heart disease, n (%)	9 (8.7)	22(10.9)	0.539
Ischaemic stroke, n (%)	8 (7.7)	12(5.9)	0.557
CHADS_2_ score	0.8 ± 1.0	0.8 ± 0.9	0.946
CHA_2_DS_2_-VASc score	1.3 ± 1.2	1.2 ± 1.2	0.599
R_2_CHADS_2_ score	1.6 ± 1.4	1.4 ± 1.3	0.082
Produce time, h	2.5 ± 1.5	2.3 ± 1.3	0.265
Exposure time, s	61.6 ± 29.3	63.3 ± 27.9	0.176
Ablation time, min	53.8 ± 17.4	55.6 ± 19.9	0.198
LAD, mm	41.6 ± 5.0	39.39 ± 3.4	< 0.001
LAV, ml	58.6 ± 17.7	50.5 ± 11.6	< 0.001
LAVI, ml/m^2^	33.7 ± 9.7	27.1 ± 6.5	< 0.001
LVEF, %	57.2 ± 5.7	59.9 ± 4.8	0.108
eGFR, ml/min/1.73^2^	53.5 ± 14.4	65.5 ± 13.3	< 0.001
CCr, ml/min	85.2 ± 26.1	101.5 ± 29.4	0.033

### Echocardiographic characteristics

**Fig. 2:**
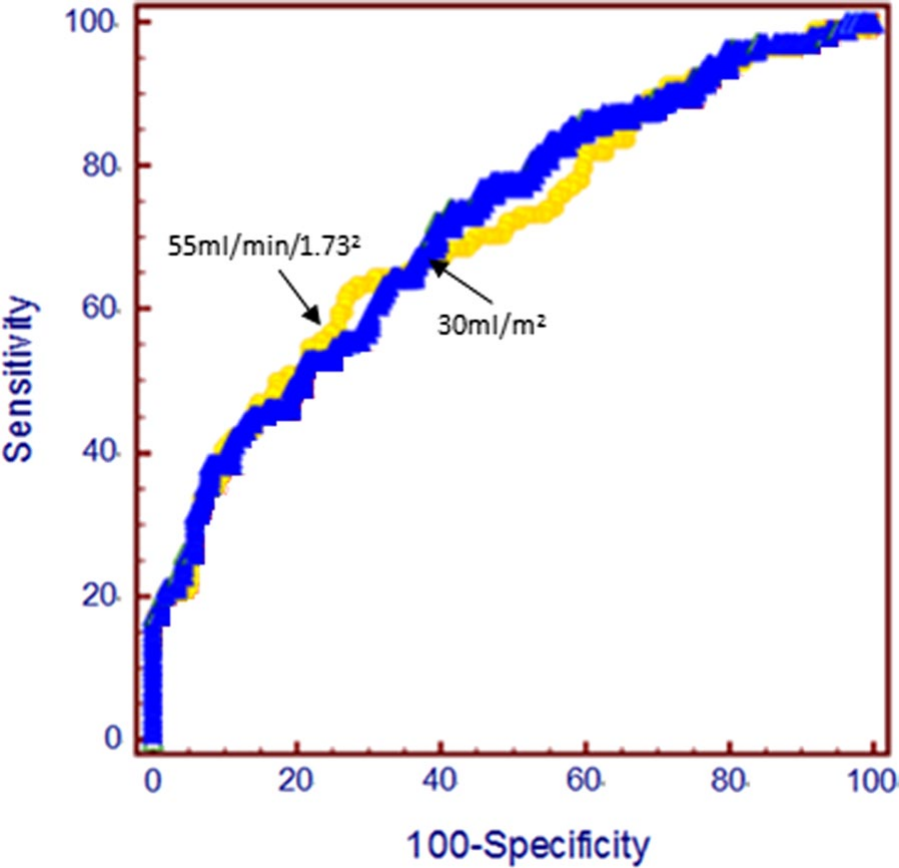
The ROC curve analysis of the eGFR (yellow line) and LAVI (blue line) according to recurrence of AF after a single ablation procedure. Arrows indicate optimal cut-off point for sensitivity and specificity

**Fig. 3:**
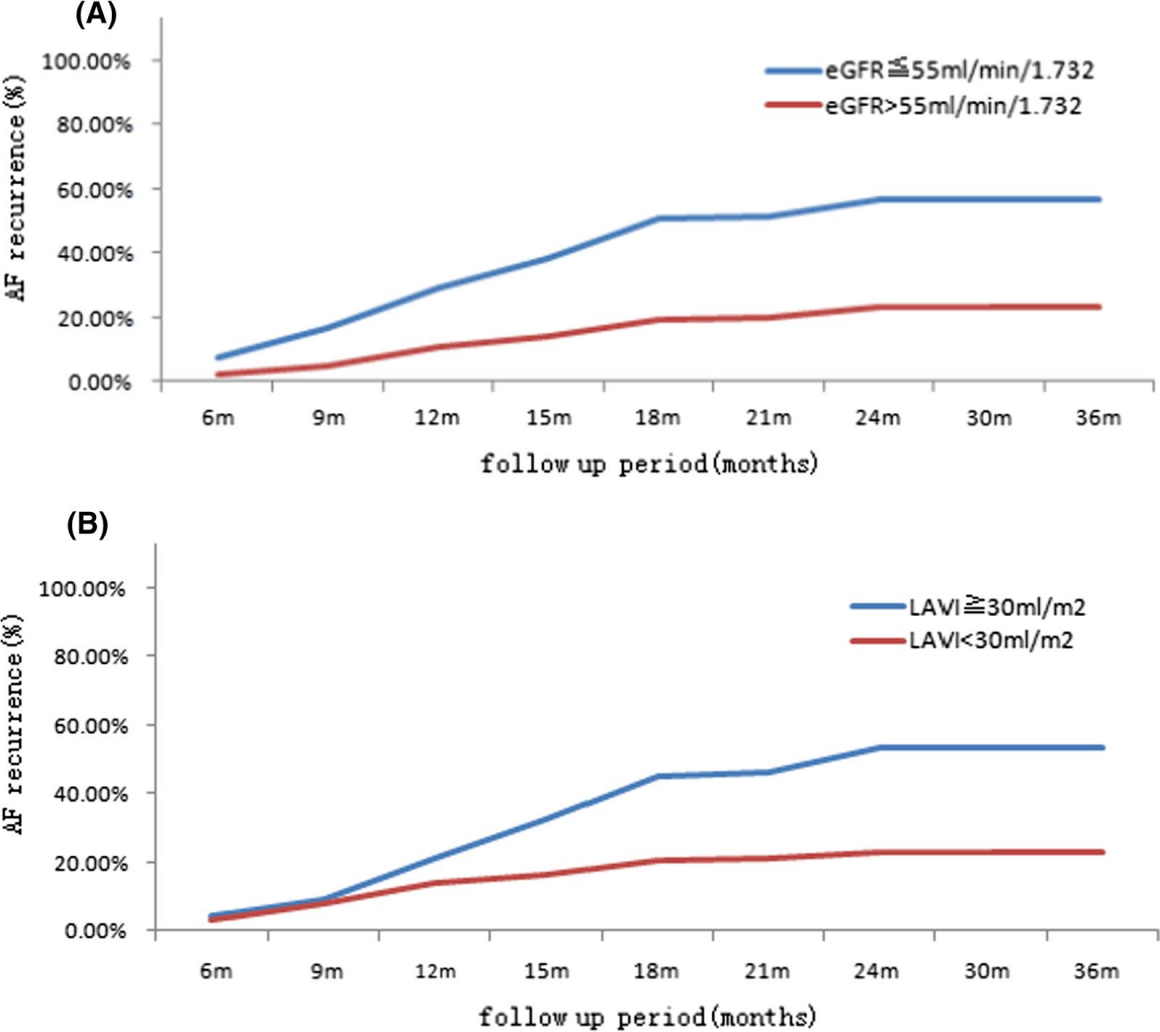
Long-term AF recurrence after a single catheter ablation: **A** the long-term recurrence in patients with or without eGFR ≤ 55 ml/min/1.73^2^. **B** The long-term recurrence in patients with or without LAVI ≥ 30 ml/m^2^

Compared with the non-recurrence group, patients in the recurrence group had a higher LAD, LAV and LAVI (Table 1). Of note, multivariate logistic analysis suggested LAVI as an independent associated factor for long-term recurrence after AF ablation (*p* < 0.0001), outperforming either LAD (*p* = 0.034) or LAV (*p* = 0.598) (Table 2). The ROC curve showed an AUC of 0.708 (95% CI, 0.65 to 0.76, *p* < 0.001) for LAVI (Fig. 2). A cut-off point of 30 ml/m^2^ of the LAVI had a specificity of 71.8% and a sensitivity of 62.5%, with a positive/negative predictive value of 52.8%/79.2% (Fig. 2, blue line). Increased LAVI contributed to higher recurrence (Fig. 3).

**Table 2:** Multivariate logistic regression analysis of predictors for recurrence after AF ablation

Variable	B	Sig	95%CI
Age	0.45	0.361	0.950–1.152
Female	− 0.815	0.314	0.936–1.169
History of AF	− 0.700	0.065	0.236–1.045
Persistent AF	− 0.841	0.218	0.113–1.645
Hypertension	− 0.997	0.528	0.017–8.145
Diabetes mellitus	− 1.962	0.292	0.224–5.401
Coronary heart disease	− 2.026	0.281	0.003–5.265
Ischaemic stroke	− 0.277	0.558	0.300–1.916
LAD	− 0.360	0.034	0.504–0.967
LAV	− 0.719	0.598	0.545–1.418
LAVI	− 0.819	0.000	0.322–0.602
eGFR	− 0.354	0.002	0.559–0.881
CCr	− 0.007	0.637	0.966–1.021

### Renal insufficiency

The patients in the recurrence group had a lower eGFR (53.5 ± 14.4 vs. 65.5 ± 13.3 ml/min/1.73^2^, *p* < 0.001) and CCr (85.2 ± 26.1 vs. 101.5 ± 29.4 ml/min, *p* < 0.05) than those in the non-recurrence group (Table 1). Multivariate logistic analysis indicated that eGFR was an independent associated factor for recurrence (Table 2). The AUC of eGFR was calculated to be 0.725 (95% CI, 0.67 to 0.77, *p* < 0.001) (Fig. 2, yellow line). The optimal cut-off point for eGFR as an independent predictor was 55 ml/min/1.73^2^, with a specificity of 79.2% and a sensitivity of 51.9% and a positive/negative predictive value of 56.2%/76.2% (Fig. 2). Patients with an eGFR ≤ 55 ml/min/1.73^2^ had a significantly increased rate of recurrence (Fig. 3).

### Independent prognostic value of eGFR and LAVI for recurrence prediction

**Fig. 4:**
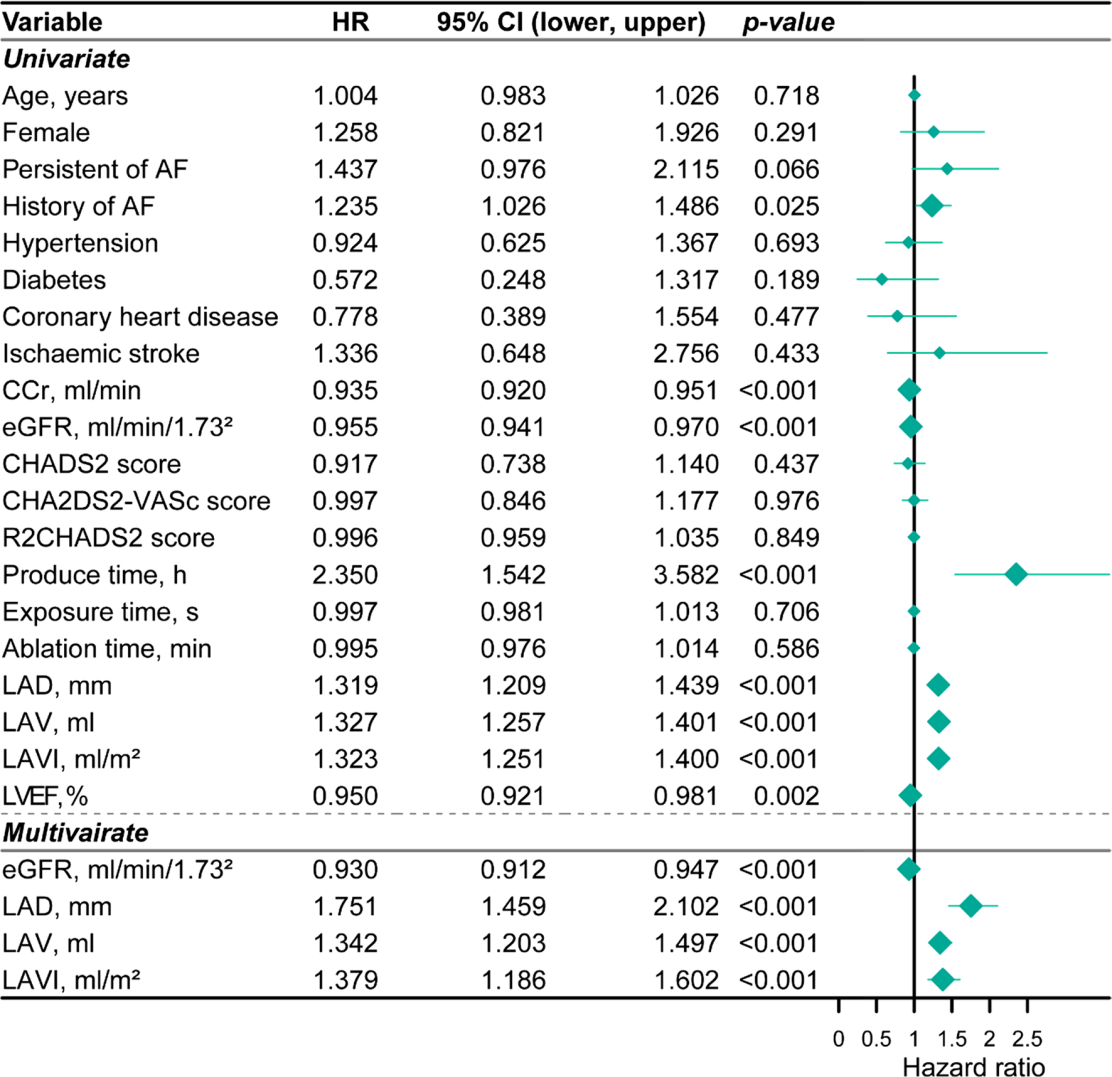
Forestplot showing univariate and multivariate Cox proportional hazard regression results considering recurrence of atrial tachyarrhythmia after catheter ablation for AF by using clinical variables

We then investigate the prognostic value of eGFR in evaluating recurrence of atrial tachyarrhythmia after catheter ablation for AF. In this manner, we considered time-to-event process and performed univariate Cox hazard proportional regression model for all clinical variables. We found history of AF (HR: 1.23, 95% CI: 1.03–1.49, *p* = 0.025), produce time (HR: 2.35, 95% CI: 1.54–1.3.58, *p* < 0.001), LAD (HR: 1.32, 95% CI: 1.21–1.44, *p* < 0.001), LAV (HR: 1.33, 95% CI: 1.26–1.40, *p* < 0.001) and LAVI (HR: 1.32, 95% CI: 1.25–1.40, *p* < 0.001) presented with risk factors for recurrence; while CCr (HR: 0.94, 95% CI: 0.92–0.95, *p* < 0.001), eGFR (HR: 0.96, 95% CI: 0.94–0.97, *p* < 0.001) and LVEF (HR: 0.5, 95% CI: 0.92–0.98, *p* = 0.002) showed significant protective effect to recurrence (Table 3, Fig. 4).

**Table 3:** Univariate and multivariate Cox regression with backward elimination of clinical variables for predicting recurrence after AF ablation

	Hazard ratio	Lower 95% CI	Upper 95% CI	*P* value
*Univariate*				
Age, years	1.004	0.983	1.026	0.718
Gender				
Male (ref.)				
Female	1.258	0.821	1.926	0.291
Persistent AF				
No (ref.)				
Yes	1.437	0.976	2.115	0.066
History of AF	1.235	1.026	1.486	0.025
Hypertension				
No (ref.)				
Yes	0.924	0.625	1.367	0.693
Diabetes				
No (ref.)				
Yes	0.572	0.248	1.317	0.189
Coronary heart disease				
No (ref.)				
Yes	0.778	0.389	1.554	0.477
Ischaemic stroke				
No (ref.)				
Yes	1.336	0.648	2.756	0.433
CCr, ml/min	0.935	0.920	0.951	< 0.001
eGFR, ml/min/1.73^2^	0.955	0.941	0.970	< 0.001
CHADS_2_ score	0.917	0.738	1.140	0.437
CHA_2_DS_2_-VASc score	0.997	0.846	1.177	0.976
R_2_CHADS_2_ score	0.996	0.959	1.035	0.849
Produce time, h	2.350	1.542	3.582	< 0.001
Exposure time, s	0.997	0.981	1.013	0.706
Ablation time, min	0.995	0.976	1.014	0.586
LAD, mm	1.319	1.209	1.439	< 0.001
LAV, ml	1.327	1.257	1.401	< 0.001
LAVI, ml/m^2^	1.323	1.251	1.400	< 0.001
LVEF, %	0.950	0.921	0.981	0.002
*Multivariate (backward elimination)*				
eGFR, ml/min/1.73^2^	0.930	0.912	0.947	< 0.001
LAD, mm	1.751	1.459	2.102	< 0.001
LAV, ml	1.342	1.203	1.497	< 0.001
LAVI, ml/m^2^	1.379	1.186	1.602	< 0.001

**Fig. 5:**
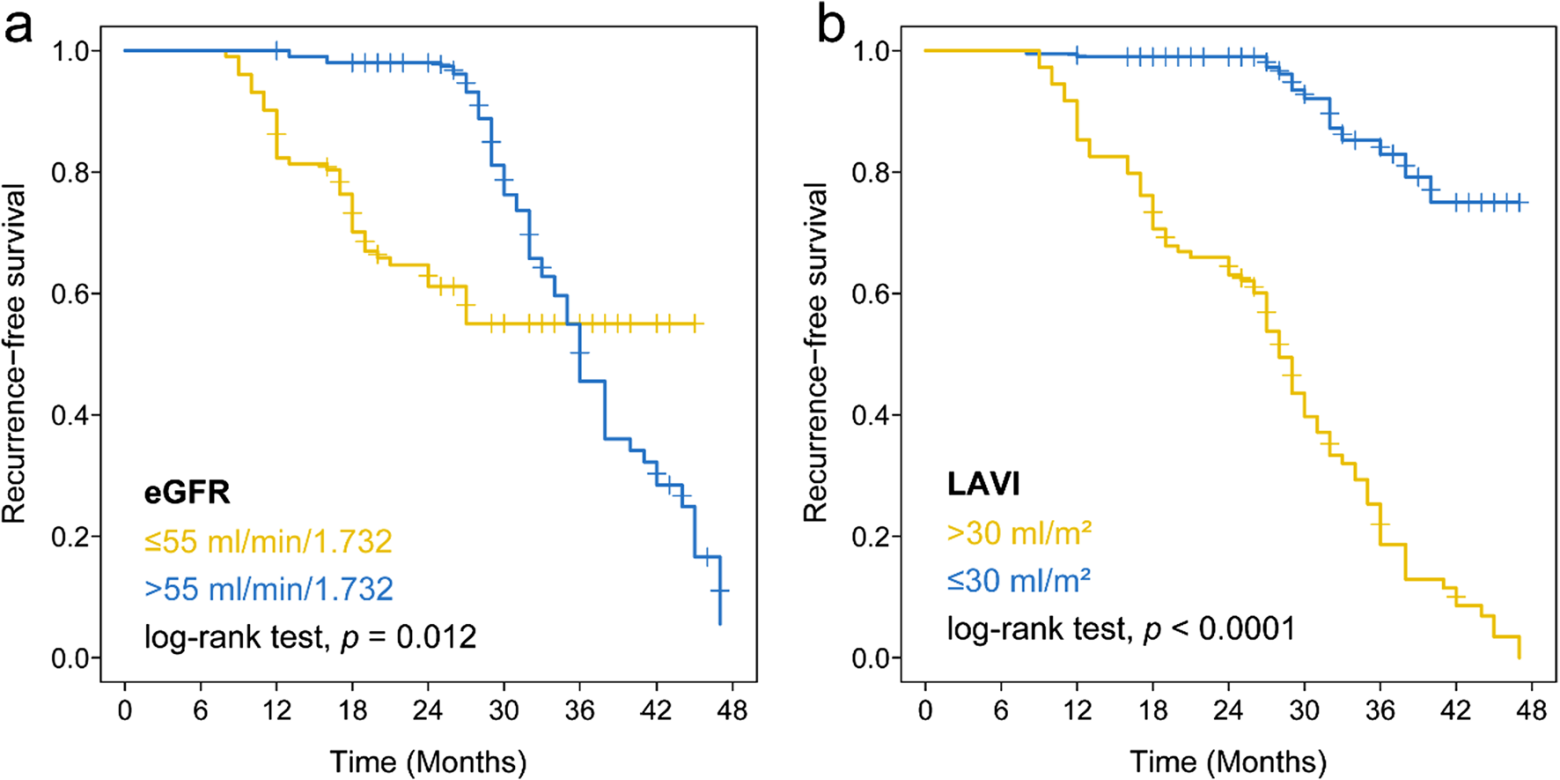
Kaplan–Meier curve of recurrence-free survival between patients with different level of **a** eGFR (ml/min/1.73^2^) and **b** LAVI (ml/m^2^)

For those variables reached statistical significance in univariate analysis, we constructed a multivariate Cox proportional hazard regression model with backward feature selection; such analysis identified four features, including eGFR (HR: 0.93, 95% CI: 0.91–0.95, *p* < 0.001), LAD (HR: 1.75, 95% CI: 1.46–2.10, *p* < 0.001), LAV (HR: 1.34, 95% CI: 1.20–1.50, *p* < 0.001) and LAVI (HR: 1.38, 95% CI: 1.19–1.60, *p* < 0.001), indicating that both eGFR and LAVI are independent prognostic factors for predicting recurrence of atrial tachyarrhythmia after catheter ablation for AF when adjusting other major clinical features (Table 3, Fig. 4). Using our pre-identified optimal cut-off, and patients with eGFR less or equal than 55 ml/min/1.73^2^ showed significant poor outcome than those with high eGFR level (log-rank test, *p* = 0.012; Fig. 5a). Likewise, patients with higher LAVI (> 30 ml/m^2^) presented unfavourable prognosis than those patients with lower LAVI level (log-rank test, *p* < 0.0001; Fig. 5b).

## Discussion

In the present study, a single ablation of AF was associated with a favourable outcome. Following up 27.2 ± 19.5 months, the overall recurrence rate was 34.0%, with 40.0% for persistent AF patients and 30.1% for paroxysmal AF patients. Multivariate logistic regression analysis indicated that pre-procedural LAVI and eGFR were independent predictors of long-term recurrence. A higher LAVI and a lower eGFR contributed to long-term recurrence after a single procedure for AF in mild to moderate renal insufficiency.

### CHADS_2,_ CHA_2_DS_2_-VASc, and R_2_CHADS_2_ score and AF

Many factors have been proposed as predictors of prognosis after AF ablation, such as congestive heart failure, hypertension, diabetes, previous stroke and sex [14–17]. CHADS_2_ and CHA_2_DS_2_-VASc scores, widely used to predict stroke risks among patients with AF, involve the abovementioned predictors. The R_2_CHADS_2_ score is a new stroke score that combines the CHADS_2_ score and an index of renal insufficiency. Chen et al. [18] demonstrated a positive relationship between new-onset AF incidence and CHADS_2_ scores in a prospective cohort study among Taiwanese patients. Further studies identified a predictive value of CHADS_2_, CHA_2_DS_2_-VASc and R_2_CHADS_2_ scores for post-ablation recurrence of AF [18–20]. Letsas KP et al. [21] reported that the predictive accuracy of both CHADS_2_ and CHA_2_DS_2_-VASc was mediocre. Inconsistent with Kornej’s and Chen’s studies, the present study showed no significant differences in CHADS_2_, CHA_2_DS_2_-VASc, or R_2_CHADS_2_ scores between the recurrence and non-recurrence groups. This finding may be partly ascribed to a rigorous inclusion. Patients with severe comorbidities were excluded. In particular, most of the patients included in this retrospective analysis were mild to moderate renal insufficiency, which had no significant effect on R_2_CHADS_2_ score.

### Renal insufficiency and AF

There is a higher prevalence of AF among patients with different stages of chronic renal disease [6]. CCr is widely used for the evaluation of renal failure in the clinic, while eGFR is preferred because it is more reliable, cheaper and easier to perform as a preoperative renal function test [22–24]. The R_2_CHADS_2_ score, involving an index of renal insufficiency determined as CCr, may not effectively evaluate the effect of renal function on the prognosis of AF ablation. The REGARDS study further demonstrated that the prevalence of AF gradually increased with a decreasing eGFR [25]. In the present study, eGFR was calculated as a renal function index instead of CCr, and multivariate logistic regression analysis showed that pre-procedure eGFR was an independent associated factor of long-term recurrence. Furthermore, multivariate Cox proportional hazard regression model indicated eGFR as an independent prognostic factors for predicting recurrence. This confirmed that eGFR was superior to CCr as a prognostic index of AF ablation. Even mild renal insufficiency may have an unavoidable effect on recurrence after AF ablation.

Active sympathetic and renin–angiotensin–aldosterone systems (RAAS) play important roles in renal insufficiency [26–28], which also involves the pathogenesis of AF [29]. Norepinephrine released from sympathetic nerve endings enhances the Ca^2+^ transient, which may activate the Na^+^–Ca^2+^ exchange current and induce late phase 3 early afterdepolarization, resulting in focal discharge and AF [30, 31]. AngII and aldosterone were elevated in patients with renal insufficiency, and both could promote oxidative stress and atrial fibrosis, so-called atrial structural remodelling [32, 33]. Aldosterone also decreases the transient outward K^+^ current and I_to_ density secondary to the rise in Ca^2+^ current, which generates abbreviation of action potential, the so-called atrial electrical remodelling, and induces AF [34]. Another workable mechanism for eGFR influencing on AF recurrence may be inflammation. Patients with renal insufficiency, even in the early stage, have been reported to have high expression of inflammatory factors, such as hypersensitive C-reactive protein, interleukin-6 and fibrinogen [35, 36]. In the early stage of renal insufficiency, inflammation could induce myocardial remodelling, which might result in recurrence of atrial arrhythmias after catheter ablation. Lin et al. [37] found that patients with higher hypersensitive C-reactive protein levels had lower mean bipolar peak voltage in the LA, suggesting extensive atrial remodelling, severe substrate and a greater possibility of non-pulmonary vein triggers. Meanwhile, these patients have a relatively higher mean dominant frequency value and widely-distributed AF nests in the LA. In addition, C-reactive protein may increase reactive oxygen species and enhance LA fibrosis, leading to atrial dilation and atrial dysfunction [38]. Therefore, the pathological mechanisms above may facilitate the recurrence of atrial tachyarrhythmia after AF ablation.

### Left atrial remodelling and AF

Water-sodium retention, hyperactive sympathetic tone and RAAS activation induced by renal insufficiency [26–28] increase LA volume overload and cause atrial remodelling. As reported in previously published studies, left atrial enlargement is the hallmark of atrial remodelling, which facilitates the prevalence of atrial arrhythmias, especially AF [39, 40]. With the enlargement of the atria, progressive changes in cellular ultrastructure and extracellular matrix (composition and volume) develop. These abnormalities induce myocardial and interstitial fibrosis, local conduction heterogeneities and electrical dissociation between muscle bundles, consequently resulting in the initiation and perpetuation of AF [39, 41]. The persistence of atrial remodelling, potentially explaining arrhythmogenic substrates, is incremental to the post-procedure recurrence of AF [42, 43].

Hui-Ling Lee et al. [44] confirmed a larger LAD was demonstrated to increase the probability of AF recurrence after surgery significantly by a three-year longitudinal study. Despite its procurability, its validity has recently been challenged, as the LA is an asymmetrical cavity. Conversely, biplane LAV provides an overall and reproducible estimation of left atrial size when compared with reference standards such as magnetic resonance imaging. Considering the individual differences, LAVI, calculated as LAV indexed to body surface area, is more comparable in accuracy and reproducibility. Marchese et al. [45] proved that LAVI was a more exact estimate of LA remodelling than LAD. LAVI was strongly associated with the risk of AF recurrence after cardioversion, with a cut-off of 31 ml/m^2^. Kataoka et al. [46] demonstrated LAVI in predicting failure of the surgical maze procedure for AF patients. However, the role of LAVI in the prognosis of AF ablation has not been well-understood.

In the present study, we found that LAD, LAV and LAVI were higher in the recurrence group. Notably, multivariate logistic analysis demonstrated that LAVI, outperforming either LAV or LAD, was an independent associated factor for predicting long-term recurrence status after catheter ablation of AF. Specifically, increased LAVI contributed to long-term recurrence, with an optimal cut-off of 30 ml/m^2^, indicating that LAVI, characterized as left atrial remodelling, may be an important determinant for the prognosis of AF ablation. Additionally, multivariate Cox proportional hazard regression also indicated that both eGFR and LAVI are independent prognostic factor for AF recurrence after a single procedure.

Recurrence after AF ablation has remained a puzzle for both doctors and patients. Evaluation of the risk factors for recurrence is crucial in boosting the success rate. In this manner, we found that a decreased eGFR and an increased LAVI had significant adverse effects in predicting long-term recurrence status after a single procedure. Therefore, the preprocedural eGFR and LAVI might be taken into consideration for optimal patient enrolment for AF ablation. Furthermore, patients with eGFR ≤ 55 ml/min/1.73^2^ or LAVI ≥ 30 ml/m^2^ may be more challenging to maintain sinus rhythm after cardioversion, and require more aggressive clinical interventions.

We acknowledged several limitations. Firstly, patients involved in this study were mainly mild to moderate renal insufficiency (CKD G2-CKD G3a) with mean eGFR 61.4 ± 14.8 ml/min/1.73^2^, and these patients with severe renal insufficiency were neglected. The current data may not sufficient to fully assess renal function in the prognosis of AF ablation. According to clinical practice, patients with severe renal insufficiency (CKD G4-5) are mostly complicated with multiple diseases, and most of them are willing to accept conservative drug-treatment. Nevertheless, increasing numbers of patients with severe renal insufficiency are likely to choose AF ablation. It is expected that the sample size will be expanded in the future to evaluate the success rate and postoperative recurrence rate of AF ablation in patients with CKD G4-5, so as to provide reference for the selection of AF treatment options in these patients. Secondly, this study is a retrospective study, all patients were treated with standard surgery, but there were still differences in surgical details among different operators, which might affect the prognosis. In addition, the collected data might be not comprehensive enough, and other important influencing factors, even those related to renal function, may also be omitted. Thirdly, there was a lack of laboratory indicators for further study of the mechanism, such as inflammatory markers, LA fibrosis and LA voltage. Fourthly, AF recurrence rates may be underestimated by ignoring asymptomatic paroxysmal AF due to the limited follow-up period. Mobile health technology may improve the comprehensive management of AF [47]. Further investigation is needed to determine whether improving renal insufficiency can enhance the long-term success of catheter ablation for AF.

## Conclusions

The present study showed that decreased eGFR and elevated LAVI contribute to increased recurrence after AF ablation. Renal insufficiency and LA remodelling might be important determinants for the long-term prognosis of AF ablation.

## Data Availability

The datasets used and/or analysed during the current study are available from the corresponding author on reasonable request.

## Abbreviations

AF: Atrial fibrillation
LA: Left atrium
LAD: Left atrial diameter
LAV: Left atrial volume
LAVI: Left atrial volume index
INR: International normalized ratio
eGFR: Estimated glomerular filtration rate
CCr: Creatinine clearance rate
CPVI: Circumferential pulmonary vein isolation
ROC: Receiver operating characteristic
AUC: Area under the curve
RAAS: Renin–angiotensin–aldosterone systems
